# Ankylosis

**Published:** 1908-04-04

**Authors:** 


### ANKYLOSIS.

The term ankylosis denotes loss of function in a joint owing to limitation or complete absence of its normal movements. The varieties of ankylosis may be classified according to whether the condition is produced by changes in the structures surrounding the joint, or in one or more of the component parts of the articulation. The former produces an immobility which is not in the true sense of the word an ankylosis, as, for instance, when gonorrhœal or rheumatic inflammation attacks the peri-articular tendons or fasciæ. Burns or scalds of the skin surrounding an articulation may lead to cicatricial contraction, which may seriously impair movements.

But these conditions are not brought to the notice of the surgeon so frequently or so prominently as the forms of ankylosis due to intra-articular disease. And here again a further sub-division may be made into (*a*) fibrous or incomplete and (*b*) osseous or complete forms. Fibrous ankylosis is caused by the contraction of diseased ligaments or by the formation of adhesions within the joint. These may stretch from point to point across the synovial membrane or may form massive bands between the diseased articular surfaces of the ends of the bones.

Limitation of movement due to synovial bands is a condition of peculiar interest to the surgeon, because, although it may be, and commonly is, the result of keeping an inflamed or injured joint at rest, it does sometimes occur in a perfectly normal joint which has been immobilised in the course of treatment for some other condition. Ankylosis, for instance, is sometimes found in one or more joints of a limb which has been kept on a splint for the treatment of a fracture. It is not possible to say in any given case how soon adhesions will form in a normal joint if the limb be kept at rest. One sees, for instance, cases of tuberculous hip treated by prolonged rest for many months, and at the end of that time there is no limitation of movement of the knee or ankle; and on the other hand it is sometimes necessary to break down adhesions in the knee or ankle after a patient has recovered from a fracture of the tibia and fibula, where the joint has only been kept at rest for a few weeks. As a general rule, one is safe in saying that a normal joint may be kept completely still for three weeks, but not longer, without any fear that adhesions will be formed in it. This is one of the reasons why it is so important to employ massage and passive movement in the treatment of fractures; for it prevents subsequent stiffness in two ways—by avoiding the formation of adhesions in the joint, and by preventing the tendons from becoming adherent to the periosteum covering the bones in the neighbourhood of the joint. When once adhesions have formed it is of course necessary to give an anæsthetic and to break them down; but even then, if they are of a considerable size they show a tendency to re-form, and a variable amount of permanent disability may be left.

Osseous or true ankylosis results from a number of causes, but the commonest are (i.) severe injuries in which the bones are crushed, and the size or shape of the callus thrown out so modifies the articular surfaces as to render movement impossible; and (ii.) acute septic or tuberculous arthritis. In either of the latter the articular surfaces are completely disorganised, and, if recovery occurs, the ends of the bones are welded together by firm bony union.

Osteoarthritis in its later stages produces a bony ankylosis, but it does so by the formation of osteophytes which grow from the ends of the bones at the edges of the articular surface; and these processes may grow to such a size that they interlock, and all movements at the joint are completely inhibited. Such ankylosis is a fairly common phenomenon when osteoarthritis attacks the vertebral column, a fact which is easily intelligible when the complex anatomy of the joints of the spine is brought to mind. It also occurs, however, in the chronic monarticular forms of the disease. It may seem a curious thing to say, but ankylosis, when it occurs in osteoarthritis, is really a blessing in disguise, because from that moment the pain which is associated with the disease disappears. The pain is produced by the movement of the disorganised surfaces one upon the other, and when this can no longer take place the patient's troubles are very much diminished. The following case presents a good example of ankylosis of the hip-joint following on osteoarthritis. The patient, a man aged 38, had noticed pain in the left hip joint for some years, particularly marked if he stooped down. Eight months before he was seen the joint seemed to him to be getting stiff, and this had gradually progressed until he was unable to flex the thigh on the abdomen at all. Since this had occurred he had had little or no pain. Twenty years previously he had had a severe injury to the same hip. He could not say exactly what had happened at the time, but he was laid up for many weeks after the accident. When he was examined it was seen that there was half an inch of shortening in the left leg, and some eversion of the foot. The left thigh was smaller in circumference than the right one by two inches, a fact which in itself was highly suggestive of joint disease. Scarpa's triangle appeared fuller on the left side, and on palpation a hard irregular mass attached to the bone could be felt. This was apparently bony in nature, and the *x*-rays showed this to be the case. Flexion and abduction were alike impossible. The probable explanation was that the original injury was an impacted extra-capsular fracture of the neck of the femur, and that osteoarthritis had supervened several years later and had led to ankylosis by interlocking of osteophytic outgrowths.

